# Perimyocarditis: An Unusual Manifestation of Dengue Virus Infection

**DOI:** 10.7759/cureus.37093

**Published:** 2023-04-04

**Authors:** Assam Ullah, Salman Khan, Asfandyar Ahmad, Muhammad Irfan, Imad Majeed

**Affiliations:** 1 Department of Internal Medicine, Khyber Teaching Hospital, Khyber Medical College, Peshawar, PAK; 2 Department of Cardiology, Khyber Teaching Hospital, Khyber Medical College, Peshawar, PAK; 3 Department of Cardiology, Hayatabad Medical Complex, Peshawar, PAK; 4 Department of Medicine, Khyber Teaching Hospital, Khyber Medical College, Peshawar, PAK

**Keywords:** dengue fever, pericarditis, myocarditis, dengue myocarditis, cardiac complications, dengue virus infection, perimyocarditis

## Abstract

Dengue is a febrile viral illness transmitted by *Aedes Aegypti* mosquito, presenting with a range of clinical features including a mild febrile illness to a life-threatening hemorrhagic fever or shock syndrome. Additionally, dengue fever can present with atypical features with the involvement of multiple organ systems including the heart. Here, we report a case of a 35-year-old female with dengue fever who presented with chest pain and dyspnea and was diagnosed with perimyocarditis.

## Introduction

Dengue virus (DENV) infection is a mosquito-borne systemic viral illness transmitted mainly via *Aedes aegypti* and *Aedes albopictus*, which are highly prevalent in tropical and subtropical regions. DENV has been estimated to cause around 390 million infections annually, of which 96 million cases manifest clinically [1]. DENV is a single-stranded RNA virus of the family *Flaviviridae* with four serotypes: DENV1, DENV2, DENV3, and DENV 4.

Most of the cases of DENV infection are asymptomatic or mild, and therefore the actual number of dengue cases is under-reported. Many cases are also mislabeled as other febrile illnesses [1]. The World Health Organization (2009) has revised the classification of dengue into two categories: dengue (with or without warning signs) and severe dengue. Severe DENV infection comprises severe plasma leakage, severe bleeding, or severe organ involvement [2]. The clinical spectrum of DENV infection is vast, ranging from asymptomatic infection to atypical presentations. Cardiac manifestations of dengue are rare, and atrioventricular blocks, atrial fibrillation, sinus node dysfunction, pericardial effusions, myocarditis, and ectopic ventricular beats have been reported during episodes of DENV infection [3].

Herein, we present a case of perimyocarditis as an unusual and atypical manifestation of DENV infection in a 35-year-old female.

## Case presentation

A 35-year-old female presented with a two-day history of acute chest pain and shortness of breath. She also came to the emergency department with high-grade fever, nausea, and myalgias five days prior to presentation and was diagnosed with dengue fever due to leukopenia, thrombocytopenia, and positive NS1 protein. At this visit, she complained of substernal chest pain for the last two days, aggravated with inspiration and lying down, and relieved by leaning forward. She complained of mild shortness of breath with normal physical activity. On general physical examination, she was alert and conscious with a blood pressure of 90/70 mmHg, a pulse of 112 beats per minute, and a respiratory rate of 20 breaths per minute with a saturation of 85% on the pulse oximeter. On cardiovascular examination, she had a pericardial rub on the left sternal border with no murmurs. She had bi-basal inspiratory crepitations on chest auscultation. Jugular venous pressure (JVP) was raised at 11 cm of water, and bilateral pitting pedal edema was noted; however, no shifting dullness was noted on abdominal examination.

**Figure 1:**
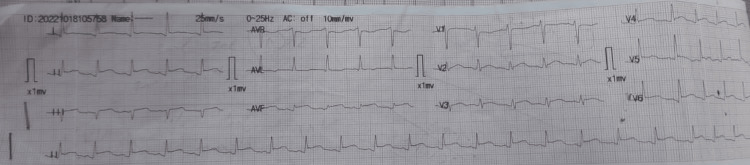
Electrocardiogram showing ST elevations and PR depressions

**Figure 2:**
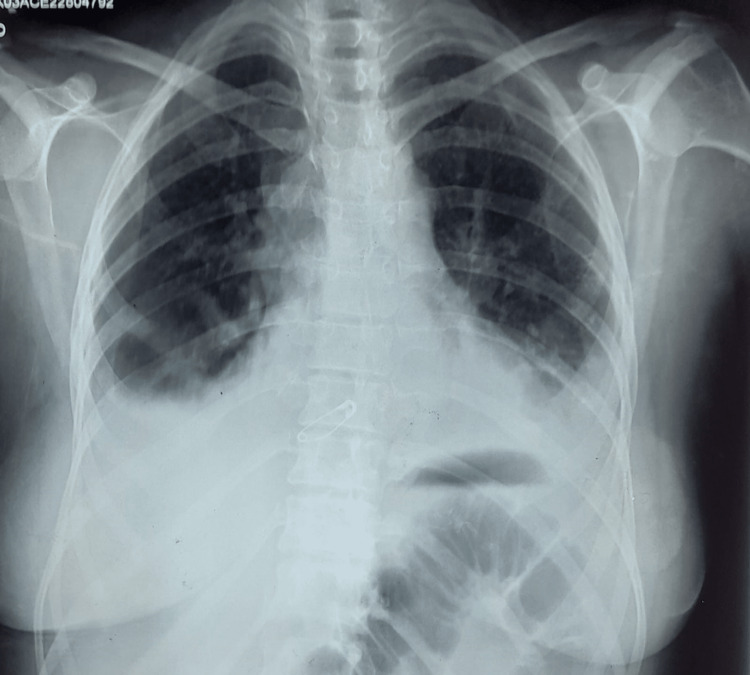
Chest X-ray showing bilateral pleural effusion

Laboratory data disclosed that her white blood cell count was 13,000/cm^3^, hemoglobin was 12.3 g/dL, mean corpuscular volume was 69 fL, hematocrit was 41% and platelet count was 1,25,000/cm^3^. Her basic metabolic panel was essentially normal except for an alanine transaminase level of 132 U/L and an alkaline phosphatase of 293 U/L. Dengue-specific antibodies and antigen testing at the previous visit revealed positive NS1 protein and reactive IgM antibody. A repeat assay revealed the same result. Her electrocardiogram (EKG) showed generalized ST elevations in most precordial and limb leads, PR depression in lead I and II, and ST/T ratio of >0.25, suggestive of pericarditis (Figure 1). Serum troponin I was raised at 1.65 ng/mL (reference range: 0-0.6 ng/mL), and arterial blood gas showed a picture of respiratory alkalosis with a pH of 7.58, pCO_2_ of 26.4 mmHg, and HCO_3_ of 24 mmol/L. Chest X-ray revealed bilateral pleural effusion (Figure 2). Lipid profile and HbA1C were within the normal range.

**Figure 3:**
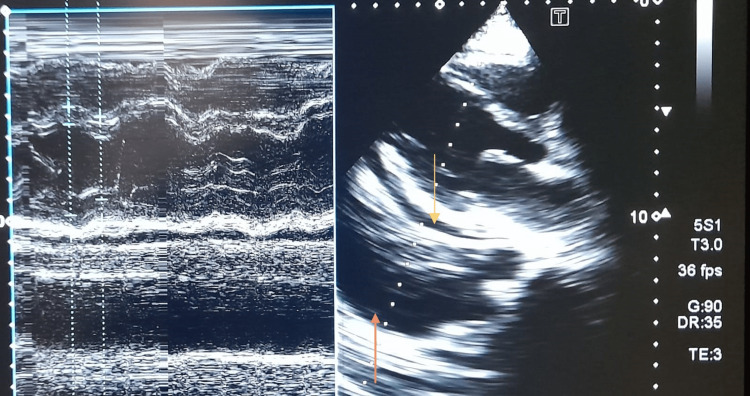
Two-dimensional echocardiography image (parasternal long axis view with M mode) showing mild pericardial effusion (yellow arrow) posteriorly and pleural effusion (orange arrow)

Two-dimensional (2D) echocardiography showed pericardial effusion, normal left ventricular (LV) size, and global hypokinesia with an LV ejection fraction of 40%, confirming the diagnosis of perimyocarditis (Figure 3). Rest of the echocardiographic findings were normal except for trace mitral and tricuspid regurgitation. She was treated with supplemental oxygen (2-5 L/min) via face mask, high-dose aspirin 600 mg three times daily, low-dose ramipril, intravenous furosemide (60 mg two times daily), and pericardiocentesis to relieve the patient's symptoms. Pericardial fluid analysis revealed cloudy fluid with raised protein and frequent polymorphonuclear leukocytes and red blood cells, but the result was inconclusive. She improved during her hospital stay within a week, as evidenced by a considerable decrease in chest crepitations and JVP. A follow-up transthoracic echocardiogram performed two weeks after admission revealed an improved LV ejection fraction of 50% with resolution of pericardial effusion, and she was discharged in stable condition.

## Discussion

Cardiac manifestations of DENV infection are rare and primarily encountered in severe infections [3]. A review involving 6,773 patients revealed sinus bradycardia as the most common cardiac abnormality (8.8%), while myocarditis and pericarditis were present in 2.9% and 0.1% of cases, respectively [4]. The risk of atypical cardiac manifestations is more in patients with non-severe dengue with warning signs and severe dengue than in patients with non-severe dengue without warning signs [5]. Dengue myocarditis/pericarditis is exceeding rare, with only a few cases reported in the world literature. Both perimyocarditis and myopericarditis have been rarely reported in DENV infections, with ejection fraction being low normal in the latter. While both terms are often used interchangeably, they represent two different disease processes. Myocarditis refers to confirmed acute pericarditis with elevated troponin but without LV systolic dysfunction. Perimyocarditis refers to acute pericarditis with elevated troponin and LV ejection fraction less than 55% [6]. Shah et al. reported a case of DENV infection in a young female, complicated by perimyocarditis and cardiac tamponade [7]. Another case of DENV infection in a young male presenting with hypotension was diagnosed as perimyocarditis later on [8]. Our case also presented with cardiovascular symptoms, i.e., chest pain and dyspnea, despite having no risk factors for any cardiac or pulmonary disease.

Common mechanisms of cardiovascular manifestations of DENV infection include endothelial dysfunction with increased permeability, systemic hypoperfusion, and hypoxemia [9]. However, the specific mechanisms responsible for atypical cardiac manifestations such as myocarditis and pericarditis have not yet been fully understood. Necrosis of myocardial fibers, perivascular inflammatory cell infiltration, and marked interstitial edema have been found on histology [10]. It is still not fully determined if dengue myocarditis results from viral invasion or is due to cytokine release associated with complement activation.

There is no evidence to support any specific treatment for DENV infection complicated by perimyocarditis apart from supportive care and frequent monitoring. Early diagnosis of myocardial involvement, controlled fluid resuscitation while avoiding overload, diuretics, and inotropic support with continuous monitoring are the cornerstones of management in dengue-affected patients with perimyocarditis [11]. Our patient presented with cardiogenic shock and hypoxia in the setting of DENV infection, with ECG and echocardiography confirming the diagnosis of perimyocarditis. Hence, there should be a high degree of suspicion of atypical cardiac manifestations in dengue patients who present with hypotension and dyspnea despite having no evidence of capillary leak syndrome.

## Conclusions

The spectrum of cardiac manifestations in DENV infection is diverse, ranging from bradycardia and subtle ST-T changes to pericarditis and fulminant myocarditis. Perimyocarditis in DENV infection is rare but can be fatal in some cases. Early diagnosis of possible cardiac involvement and prompt recognition and management of hemodynamic instability are vital in preventing morbidity and mortality in DENV infection complicated by perimyocarditis.
